# Supplementary material for: Cell-Free DNA as a Biomarker at Diagnosis and Follow-Up in 256 B and T-Cell Lymphomas
Source: Cancers (Basel). 2024 Jan 11;16(2):321. doi: 10.3390/cancers16020321 (PMC10813584; doi:10.3390/cancers16020321)

Supplementary Materials

Supplementaty Table S1. Plasma cfDNA concentration (ng/mL) according to clinical features. N: 249. AA: Ann Arbor; LDH: lactate dehydrogenase; B2-MG: beta-2 microglobulin; BM: bone marrow. U-Mann-Whitney test, with exact Fisher correction when applicable.

	Median (IQR)	p
**Gender**		
**Male**	31.2 (14.7-59.6)	0.623
**Female**	25.5 (13.0-59.1)	
**Age**		
**<60 years**	25.3 (12.7-49.3)	0.137
**> 60 years**	30.0 (15.2-63.6)	
**LDH**		
**Normal**	23.8 (12.8-43.2)	<0.0001
**Elevated**	51.0 (24.4-85.7)	
**B2-MG**		
**Normal**	24.5 (13.3-47.6)	<0.0001
**Elevated**	38.2 (21.8-72.8)	
**AA stage**		
**I-II**	25.5 (14.5-47.1)	0.020
**III-IV**	30.0 (14.6-63.6)	
**B symptoms**		
**No**	25.6 (14.4-65.8)	0.001
**Present**	42.8 (18.5-81.8)	
**BM infiltration**		
**No**	30.4 (15.0-59.6)	0.716
**Present**	23.3 (13.1-55.8)	

Supplementary Table S2. Comparison of cfDNA according to lymphoma subtype vs controls. LBCL: large B-cell lymphoma; FL: follicular lymphoma; MZL: marginal zone lymphoma; MCL: mantle cell lymphoma; small lymphocytic lymphoma/chronic lymphocytic leukemia; LpL/W: lymphoplasmacytic lymphoma/Waldenstöm macroglobulinemia; LPS-NOS: circulating low-grade unclassifiable B-cell lymphoma; cHL: classic Hodgkin lymphoma; AITL: angioimmunoblastic T-cell lymphoma; TCL: T-cell lymphoma. *1 localized BL patient was not considered.

Type of lymphoma	N	p
**LBCL**	88	<0.001
**FL**	47	<0.001
**MZL**	30	<0.001
**MCL**	13	<0.001
**SLL/CLL**	7	<0.001
**LpL/WM**	5	0.012
**LPS-NOS**	6	<0.001
**cHL**	30	<0.001
**AITL**	9	<0.001
**Other TCL**	6	<0.001
**Others**	6	0.002
**All cases**	248*	<0.001

Supplementary Table S3. *P* values for associations of cfDNA levels with characteristics at presentation according to the main lymphoma subtypes. AA: Ann Arbor; LDH: lactate dehydrogenase; B2-MG: beta-2 microglobulin; BM: bone marrow; IPI: international prognostic index; FLIPI: follicular international prognostic index. Spearman test for age, LDH, B2-MG; U-Mann-Whitney test or Kruskal Wallis test for gender, stage, B-symptoms, BM involvement, IPI and FLIPI.

	**LBCL (N=88)**	**FL (N=47)**	**MZL (N=30)**	**MCL (N=14)**	**cHL (N=30)**
**Age**	0.300 (r=0.111)	**0.017** (r=0.348)	0.054 (r=0.777)	0.462 (r=0.214)	**<0.0001** (r=0.611)
**LDH**	**0.003** (r=0.316)	0.271 (r=0.168)	**0.016** (r=0.443)	0.516 (r=0.190)	0.949 (r=0.012)
**B2-MG**	**0.002** (r=0.329)	0.200 (r=0.199)	0.066 (r=0.419)	0.852 (r=0.055)	0.056 (r=0.359)
**Gender**	0.232	0.203	0.595	0.791	0.154
**AA stage**	0.090	0.601	0.072	NA	0.179
**B-symptoms**	**0.001**	0.230	0.901	1	0.790
**BM involvement**	0.134	0.515	0.094	0.264	0.705
**IPI**	**0.0006**	**0.012**	**0.044**	0.066	NA
**FLIPI**	-	0.156	-	-	-

Supplementary Table S4. Clinical features of LBCL patients at diagnosis. AA: Ann Arbor; LDH: lactate dehydrogenase; B2-MG: beta-2 microglobulin; BM: bone marrow; IPI: international prognostic index.

	LBCL (N=49)
**Age, years (range)**	62 (20-86)
**Gender**	
**Male**	55%
**Female**	45%
**ECOG PS**	
**0-1**	84%
**2-4**	16%
**AA stage**	
**I-II**	47%
**III-IV**	53%
**B symptoms**	33%
**LDH elevated**	59%
**B2-MG elevated**	27%
**BM involvement**	6%
**IPI**	
**Low**	29%
**Low/Intermediate**	37%
**Intermediate/High**	24%
**High**	10%

Supplementary Table S5. Associations of cfDNA with clinical characteristics at diagnosis in de LBCL cohort. *1 patient without available cfDNA. AA: Ann Arbor; LDH: lactate dehydrogenase; B2-MG: beta-2 microglobulin; BM: bone marrow; IPI: international prognostic index.

	LBCL (N=48*) p
**Age**	0.241 (r=0.173)
**LDH**	0.138 (r=0.349)
**B2-MG**	0.194 (r=0.191)
**Gender**	0.582
**AA stage**	0.858
**B-symptoms**	0.498
**ECOG**	0.710
**BM involvement**	0.134
**IPI**	**0.015**

Supplementary Figure S1. Scattered plot showing correlations between cfDNA and LDH and beta 2-microglobulin (B2-MG) (N:249).

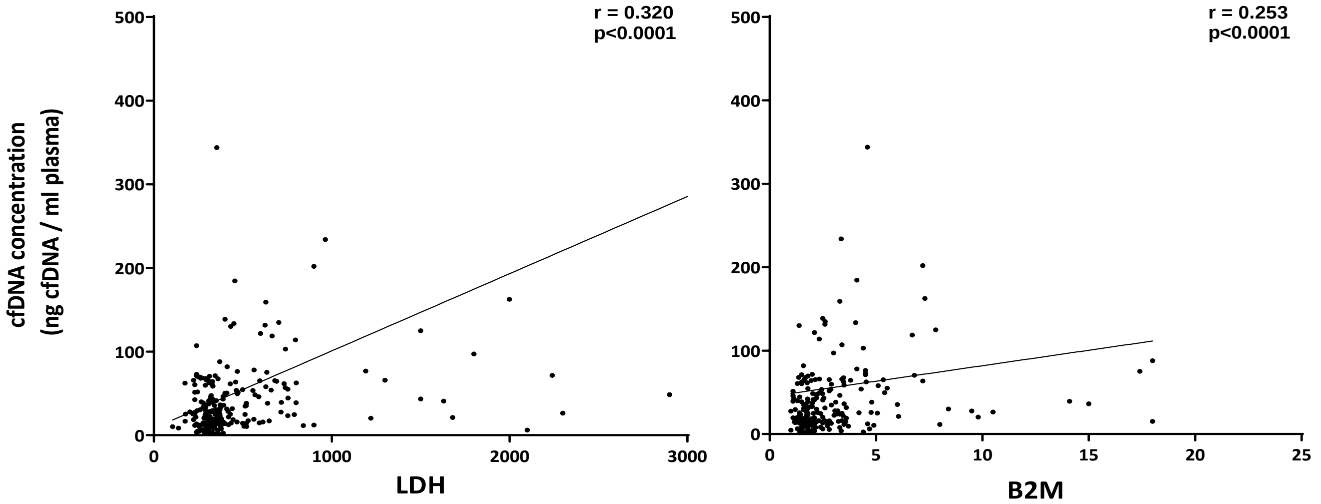

Supplementary Figure S2. PFS and OS based on the ctDNA kinetics (reduction of >2.5 log hGE/mL or < 2.5 log hGE/mL from diagnosis to the end of treatment).

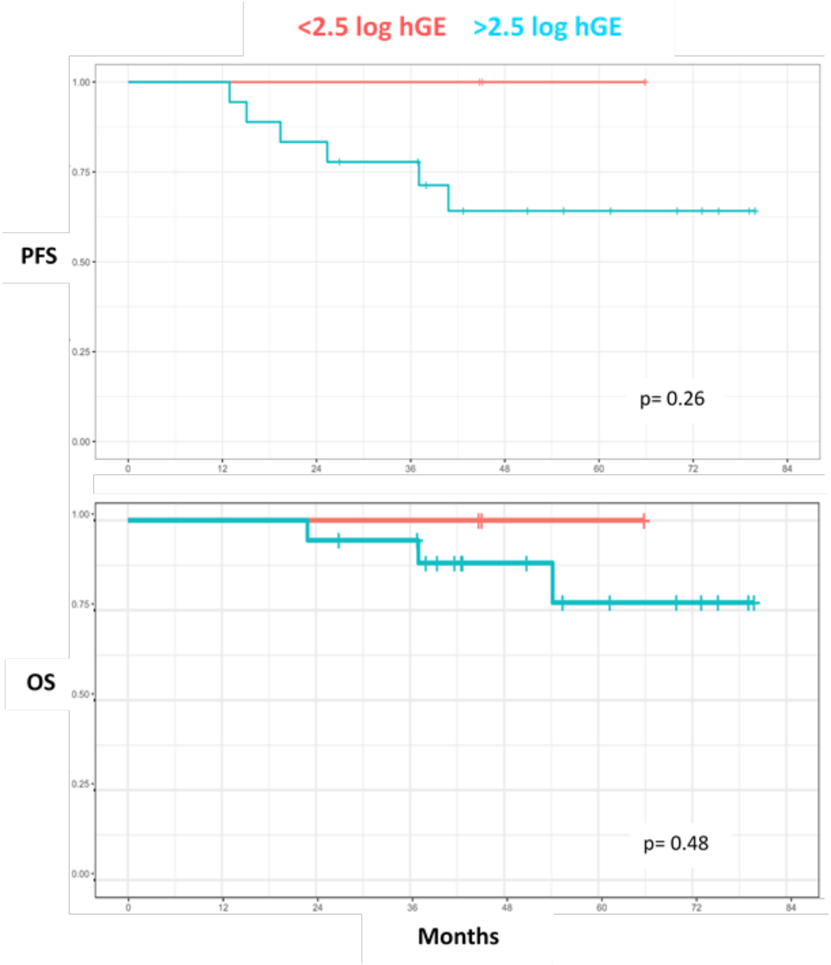

Supplement: Supplementary file 1 [file cancers-16-00321-s001.zip › cancers-2791187-supplementary.pdf]
